# Genome-wide identification, characterization, expression and enzyme activity analysis of coniferyl alcohol acetyltransferase genes involved in eugenol biosynthesis in *Prunus mume*

**DOI:** 10.1371/journal.pone.0223974

**Published:** 2019-10-16

**Authors:** Tengxun Zhang, Tingting Huo, Anqi Ding, Ruijie Hao, Jia Wang, Tangren Cheng, Fei Bao, Qixiang Zhang

**Affiliations:** 1 Beijing Key Laboratory of Ornamental Plants Germplasm Innovation & Molecular Breeding, Beijing Forestry University, Beijing, China; 2 National Engineering Research Center for Floriculture, Beijing Forestry University, Beijing, China; 3 Beijing Laboratory of Urban and Rural Ecological Environment, Beijing Forestry University, Beijing, China; 4 Key Laboratory of Genetics and Breeding in Forest Trees and Ornamental Plants of Ministry of Education, Beijing Forestry University, Beijing, China; 5 School of Landscape Architecture, Beijing Forestry University, Beijing, China; Universidad Politecnica de Cartagena, SPAIN

## Abstract

*Prunus mume*, a traditional Chinese flower, is the only species of *Prunus* known to produce a strong floral fragrance, of which eugenol is one of the principal components. To explore the molecular mechanism of eugenol biosynthesis in *P*. *mume*, patterns of dynamic, spatial and temporal variation in eugenol were analysed using GC-MS. Coniferyl alcohol acetyltransferase (CFAT), a member of the BAHD acyltransferase family, catalyses the substrate of coniferyl alcohol to coniferyl acetate, which is an important substrate for synthesizing eugenol. In a genome-wide analysis, we found 90 *PmBAHD* genes that were phylogenetically clustered into five major groups with motif compositions relatively conserved in each cluster. The phylogenetic tree showed that the PmBAHD67-70 proteins were close to the functional CFATs identified in other species, indicating that these four proteins might function as CFATs. In this work, 2 *PmCFAT* genes, named *PmCFAT1* and *PmCFAT2*, were cloned from *P*. *mume* ‘Sanlunyudie’, which has a strong fragrance. Multiple sequences indicated that PmCFAT1 contained two conserved domains, HxxxD and DFGWG, whereas DFGWG in PmCFAT2 was changed to DFGFG. The expression levels of *PmCFAT1* and *PmCFAT2* were examined in different flower organs and during the flowering stages of *P*. *mume* ‘Sanlunyudie’. The results showed that *PmCFAT1* was highly expressed in petals and stamens, and this expression increased from the budding stage to the full bloom stage and decreased in the withering stage, consistent with the patterns of eugenol synthesis and emission. However, the peak of gene expression appeared earlier than those of eugenol synthesis and emission. In addition, the expression level of *PmCFAT2* was higher in pistils and sepals than in other organs and decreased from the budding stage to the blooming stage and then increased in the withering stage, which was not consistent with eugenol synthesis. Subcellular localization analysis indicated that PmCFAT1 and PmCFAT2 were located in the cytoplasm and nucleus, while enzyme activity assays showed that PmCFAT1 is involved in eugenol biosynthesis in vitro. Overall, the results suggested that *PmCFAT1*, but not *PmCFAT2*, contributed to eugenol synthesis in *P*. *mume*.

## Introduction

Floral scents play important and significant roles as attractants for pollinators and defence compounds against animals and microorganisms [1, 2]. In addition, floral scents can raise the aesthetic value of ornamental plants and benefit human health [3]. However, many plants have lost their fragrance because breeding objectives were focused on flower type, colour, long vase life and so on [4]. In recent years, more breeders have attached importance to flower fragrance.

*Prunus mume* Sieb. et Zucc. is a famous traditional Chinese ornamental tree with a long history of cultivation and emits a recognizable pleasant floral scent unlike other *Prunus* species [5]. Studies have shown that eugenol is the most important constituent of the characteristic floral scent of *P*. *mume* [6]. Eugenol, with the exotic smell of cloves, is among the volatile phenylpropanoid compounds derived from phenylalanine [7]. It is often synthesized in vegetables to defend against herbivores and pathogens and attract pollinators [2]. Although there is little eugenol in these plants, its odour perception threshold is low, making it possible for this scent to be the characteristic scent [8].

Eugenol is formed by the action of eugenol synthase (EGS) on the substrate coniferyl acetate, which is synthesized from coniferyl alcohol and acetyl-CoA in a reaction catalysed by coniferyl alcohol acetyltransferase (CFAT) [9]. Coniferyl alcohol is not only a precursor of coniferyl acetate but also a type of monolignol involved in the synthesis of lignin. The biochemical steps before the monolignol branch point are shared between phenylpropanoids and lignin during biosynthesis [10]. Thus, we hypothesized that CFAT is a key enzyme separating eugenol metabolism from lignin metabolism, which has important significance for the synthesis of eugenol. To date, most studies on the biosynthesis of eugenol and isoeugenol have mainly focused on the cloning and analysis of *EGS* and *IGS*, with a total of 18 *EGS/IGS* genes isolated from different plants [11, 12], whereas few have focused on *CFAT*. CFAT is a member of the BAHD acyltransferase family [9], with two conserved motifs, HxxxD and DFGWG. Only one coniferyl alcohol acyltransferase, *PhCFAT*, has been characterized from petunia hybrids, and RNAi suppression of this gene resulted in inhibition of isoeugenol biosynthesis [9]. Two *Larrea* cinnamyl alcohol acyltransferases, LtCAAT1 and LtCAAT2, isolated from *Larrea tridentata* catalysed the production of coniferyl acetate using coniferyl alcohol as a substrate. Purified LtCAAT1 *in vitro* could catalyse more substrates than PhCFAT, but coniferyl alcohol was the preferred substrate overall [13].

*P*. *mume* is the only species of *Prunus* known to produce a strong floral fragrance. Most interspecific hybrids between *P*. *mume* and other species of *Prunus* lack this fragrance [6, 14], in which eugenol is a principal component contributing to the floral scent among both emitted and endogenous compounds. To solve this breeding problem, we must understand the molecular mechanism of eugenol production. Here, 90 *PmBAHD* genes in *P*. *mume* were screened in a genome-wide analysis, and detailed phylogenetic analyses and motif construction were performed. Finally, by combining the amounts of eugenol from different organs and during flowering stages, 2 *PmCFAT* genes were isolated and analysed from ‘Sanlunyudie’, which features a typical floral scent of *P*. *mume*. This study aimed to clarify the most likely *PmCFAT* genes functioning in eugenol biosynthesis and to provide a better understanding of the biosynthesis of floral scents in *P*. *mume*.

## Materials and methods

### Plant materials

**Fig 1:**
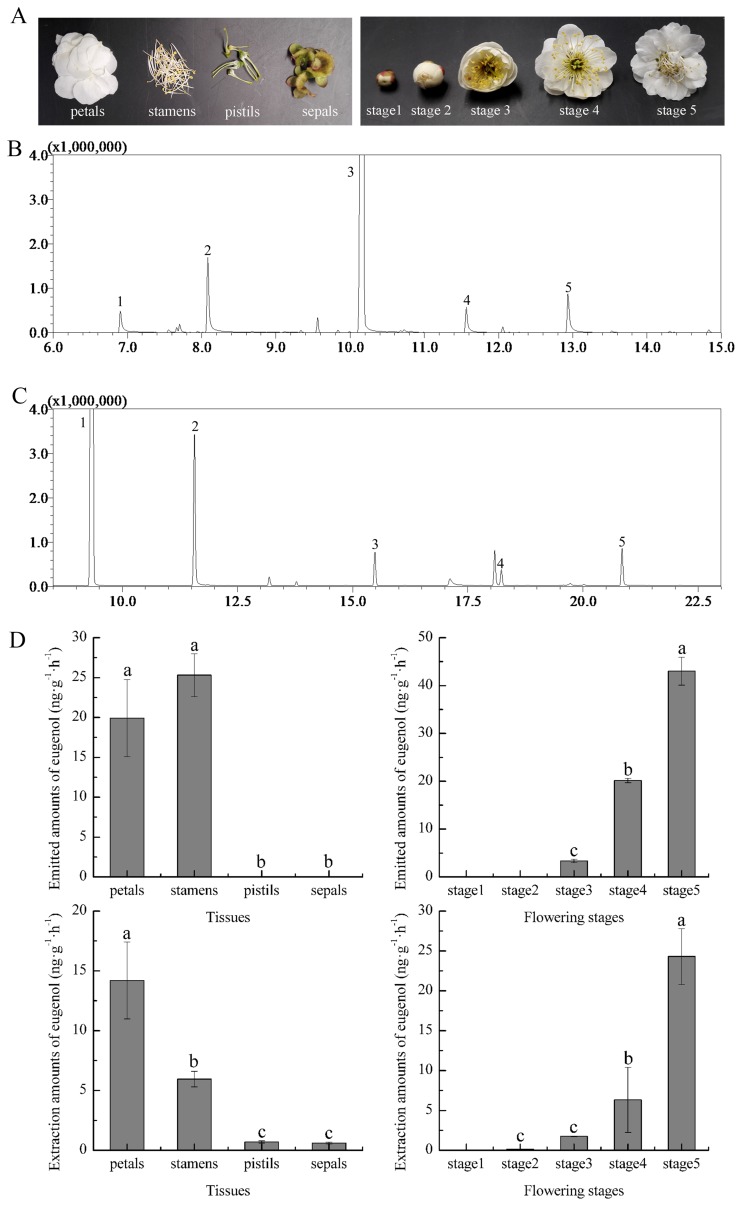
Spatial and temporal analyses of eugenol. (A) Petals, stamens, pistils, and sepals of a flower and different flowering stages. (B) Emitted compound analysis of the flowers of *P*. *mume*. (C) Extraction compound analysis of the flowers of *P*. *mume*. 1, Benzaldehyde; 2, Benzyl alcohol; 3, Benzyl acetate; 4, Benzyl propionate; 5, Eugenol. (D) The absolute amounts of volatile compounds and endogenous eugenol from different flower organs and flowering stages of *P*. *mume*.

*P*. *mume* ‘Sanlunyudie’, with a full pleasant floral scent, was chosen for study. The tested materials at different developmental stages and various tissues were collected from the campus of Beijing Forestry University. The different floral organs included petals, stamens, sepals and pistils. Flower development was classified into five stages, i.e., flowers with tightly closed buds (stage 1), flowers with slightly open buds (stage 2), flowers starting to open (stage 3), flowers in full bloom (stage 4), and completely wilted flowers (stage 5) (Fig 1A).

Tobacco plants (*Nicotiana benthamiana*) used for subcellular localization were grown under standard glasshouse conditions (16-h photoperiod, 60–65% humidity and day/night temperatures of 22/17°C).

### Emitted and endogenous eugenol collection and gas chromatography–mass spectrometry (GC–MS) analysis

The methods used for the analysis of emitted and extraction compounds were different. The emitted volatile compounds were collected in a 20 ml headspace bottle, and the endogenous compounds were extracted through ethyl acetate as in a previous study [15]. Then, 2.5 ng benzyl propionate was added as an internal standard. The floral volatile compounds were detected using GC-MS. To detect the emitted volatile compounds, the chromatography programme started at 40°C for 2 min, and the temperature was increased to 200°C at a rate of 5°C /min and held for 6 min. The GC-MS programme for endogenous compound analysis was performed as in a previous study [14]. The volatile compounds from each sample were measured in triplicate.

### Database searching and identification

To obtain the candidate CFATs in *P*. *mume*, the *P*. *mume* genome v.1.0 (http://prunusmumegenome.bjfu.edu.cn/) was searched using BlastP with the protein sequences of PhCFAT (GenBank accession No. DQ767969), LtCAAT1 (GenBank accession No. KF543260) and LtCAAT2 (GenBank accession No. KF543261) as query sequences and an e-value of 1e^-5^. All putative BAHDs were further screened to confirm the presence of the conserved domains HxxxD and DFGWG by multiple alignment using DNAMAN 7.0.

### Phylogenetic and conserved motifs analyses

Sequences of putative CFATs of other species were downloaded from the NCBI protein database (http://www.ncbi.nlm.nih.gov/protein/). The phylogenetic tree was constructed with ClustalX 2.0 [16] software and MEGA 7 [17] based on the neighbour joining (NJ) method with 1000 bootstrap replicates. Conserved motifs of candidate CFATs of *P*. *mume* were analysed using the Multiple EM for Motif Elicitation (MEME) programme (http://meme-suite.org/tools/meme) with the following parameters: the number of repetitions was random, the maximum number of motifs was set to identify 20 motifs, and the optimum motif width was set to 5–60.

### Transcriptome dataset analyses

The transcriptomes of bud and flower were obtained for comparison from the Genome Sequence Archive (GSA) with the accession numbers PRJCA000274. The different organ (flower, fruit, leaf, root, and stem) data sets were acquired from the NCBI Sequence Read Archive (SRA) with accession number SRP014885. A heat map illustrator (HemI_1.0) was used to draw the heat map with the default values [18].

### RNA extraction and cloning of *P*. *mume* CFATs

Total RNA from all tissues was extracted following the instructions from the manual of the EASYspin Plus Plant RNA Extraction Kit (Aidlab, Beijing, China). The first-strand cDNA was transcribed using a TIANScript RT Kit (KR107, Tiangen, Beijing, China). The flowers at the full bloom stage (1 day after opening) were used as plant material for gene cloning. The specific primers were designed by combining Primer 5 with a website (http://www.idtdna.com/calc/analyzer). One pair of primers, *PmCFATIF* and *PmCFATIR*, was designed to amplify the cDNA sequences of *PmBAHD67-69* with the following PCR programme: 95°C for 5 min, 30 cycles of 98°C for 10 s, 51°C for 15 s, and 72°C for 1.5 min; and a final step at 72°C for 10 min. Then, the products were stored at 4°C. The sequence of *PmBAHD70* was obtained through touchdown PCR protocols with the primers *PmCFATIIF* and *PmCFATIIR*. The amplification procedure was as follows: pre-denaturation at 95°C for 5 min, 15 cycles of denaturation at 98°C for 10 s, annealing at 63°C for 30 s, extension at 72°C for 1.5 min; 20 cycles of denaturation at 98°C for 10 s, annealing at 57°C for 30 s, extension at 72°C for 1.5 min; and holding at 72°C for 10 min. The sequences of the primers are listed in S1 Table.

### *PmCFAT1* and *PmCFAT2* expression analyses by quantitative real-time PCR

Total RNA was extracted from five developmental stages and 4 flower organs, separately, with the methods previously described. Each sample was taken from at least three flowers, and the three sample pools served as the three biological replicates; for each replicate, quantitative real-time PCR (qRT-PCR) was performed thrice. After removing residual DNA, 1 μg RNA was reverse transcribed into cDNA using the PrimeScript RT Reagent Kit with gDNA Eraser (Perfect Real Time) (TaKaRa, Dalian, China) according to the manufacturer’s instructions. We used two pairs of primers, *PmCFAT1-qRT-F* and *PmCFAT1-qRT-R*, to amplify *PmCFAT1* and *PmCFAT2-qRT-F* and *PmCFAT2-qRT-R* for *PmCFAT2*. The *PmPP2A* gene served as an internal standard [19]. The primers are presented in S1 Table. QRT-PCR was performed on the PikoReal Real-Time PCR System (Thermo Fisher Scientific, China) with SYBR Premix Ex Taq II (TaKaRa). The programme was as follows: 95°C for 30 s, 40 cycles of 95°C for 5 s and 60°C for 30 s, and 60°C for 30 s. The relative transcript levels were calculated using the 2^-ΔΔ*Ct*^ method.

### Transient expression and subcellular localization of PmCFATs in tobacco

Subcellular localization was predicted by WoLF PSORT (http://wolfpsort.seq.cbrc.jp/). The full-length cDNA with added restriction enzyme sites (*Xba* I/*Kpn* I) of *PmCFAT1* and *PmCFAT2* was amplified using *PmCFAT1*,*2*-transF and *PmCFAT1*,*2*-transR. The expression vector *pSuper1300*::*GFP* was linearized by restriction enzyme digestion with *Xba* I and *Kpn* I. These two fragments were connected by T4 ligase (Takara, Dalian, China), and the recombinant plasmid was confirmed by restriction enzyme digestion of *XbaI and KpnI*. The constructed plasmids, *pSuper1300*::*PmCFAT1*::*GFP* and *pSuper1300*::*PmCFAT2*::*GFP*, were injected into the leaves of 6-week-old tobacco (*N*. *benthamiana*) through *Agrobacterium tumefaciens* EHA105-mediated agro-infiltration, with the empty vector *pSuper1300*::*GFP* as a control. After infection for 48–56 h, the green fluorescence protein (GFP) was observed under the Leica TCS SP8 laser scanning confocal microscope (Leica, Germany) using labelled pieces of tobacco leaves placed on glass slides.

### Enzyme activity of PmCFAT proteins *in vitro*

The infected leaves overexpressing *PmCFAT1*, *PmCFAT2* and the empty vector were collected and placed in liquid nitrogen. Total crude proteins were extracted from the leaves using RIPA lysis buffer with a protease inhibitor cocktail (Roche). Two hundred micrograms of crude proteins were added to 2 mL of enzyme reaction buffer containing 50 mM Tris-HCl (pH 7.2), 0.01 mM MgCl_2_, 0.5 mM acetyl-CoA, 1 mM coniferyl alcohol and 1 mM DTNB. The assays were carried out in a 30°C water bath for 1 h, and the increase in absorbance at 412 nm was recorded using a spectrophotometer to detect the production of 2-nitro-5-thiobenzoic acid by the reaction of DTNB with free CoA [15]. The components were extracted with 0.5 mL ethyl acetate. Then, 2.5 ng benzyl propionate was added as an internal standard, and eugenol was detected using GC-MS. The enzyme activity was calculated as the value of the increase in absorbance in 1 min.

## Results

### Spatial and temporal analyses of eugenol in *P*. *mume*

Eugenol is one of the main components contributing to the floral scent of *P*. *mume*. The emitted and endogenous eugenol from fresh flowers of *P*. *mume* ‘Sanlunyudie’, with a strong fragrance, were analysed using GC-MS (Fig 1B and 1C). Generally, eugenol is emitted mainly from petals and stamens. As shown in Fig 1D, eugenol was not emitted significantly differently from the petals and stamens. Similarly, the extracted eugenol amounts in the petals and stamens were higher than those in the other two organs, although the eugenol content was higher in petals than in stamens. During flower development, for both emitted and endogenous eugenol, minimal eugenol was detected in stage 1 and then increased from stage 1 to stage 5, at which point maximum release occurred (Fig 1D).

### Identification and gene structures of *PmCFAT* in *P*. *mume*

To identify the *CFAT* genes of *P*. *mume*, the protein sequences of PhCFAT, LtCAAT1nd LtCAAT2 were used as queries to search the genome database of *P*. *mume*. A total of 90 putative *CFAT-like* genes identified in the *P*. *mume* genome are listed in S1 Table, including the previously reported PmBEAT family [15]. All the putative CFAT-like protein sequences had the typical characteristics of the BAHD family, so we named these genes *PmBAHDs*. All the identified *PmBAHD* genes encoded proteins ranging from 287 to 664 amino acids long, with molecular weights ranging from 32.25 kDa to 73.27 kDa. Most of the candidate genes (72.2%) had no introns, while another 23.3% contained 1 intron, 3 genes contained 2 introns, and 1 gene contained 4 introns. Although the DFGWG site was conserved in members of the BAHD family, this conserved domain may be mutated in some proteins, as indicated by as many as 16 types of sequence changes in *Arabidopsis* [20]. Twelve pattern types were found for this domain in the amino acid sequences of the candidate PmBAHDs, such as DFGWG, NFGWG, and SFGWG.

### Phylogenetic and conserved motif analyses of the PmBAHD family

**Fig 2:**
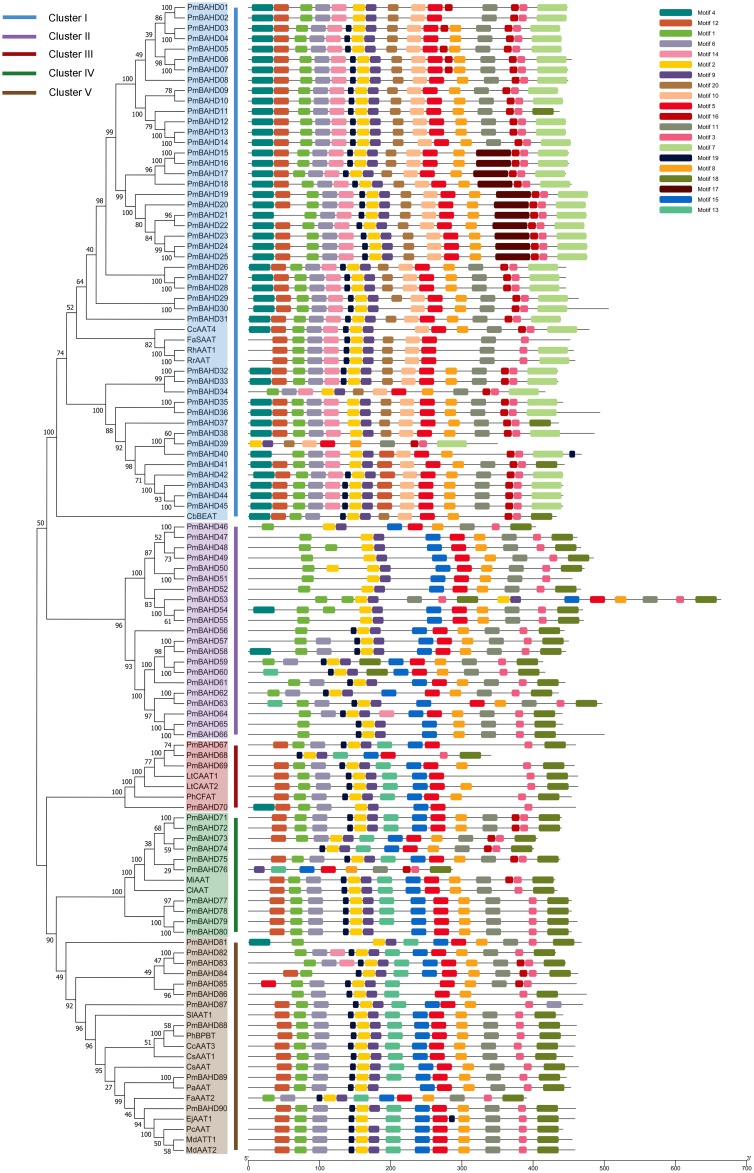
Phylogenetic tree and motif compositions of putative PmBAHDs from *P*. *mume* and related proteins. GenBank accession number: PhCFAT (*Petunia hybrida*, ABG75942.1), LtCAAT1 (*Larrea tridentata*, AHA90802.1), LtCAAT2 (*Larrea tridentata*, AHA90803.1), PhBPBT (*Petunia hybrida*, AAU06226.1), CbBEAT (*Clarkia breweri*, AAC18062.1), PaAAT (*Prunus armeniaca*, ACF07921.1), MdATT1 (*Malus domestica*, AAU14879.2), MdAAT2 (*Malus domestica*, AAS79797.1), FaAAT2 (*Fragaria* x *ananassa*, AEM43830.1), FaSAAT (*Fragaria* x *ananassa*, AAG13130.1), *PcAAT* (*Pyrus communis*, AAS48090.1), EjAAT1 (*Eriobotrya japonica*, AHC32224.2), RhAAT1 (*Rosa hybrid cultivar*, AAW31948.1), RrAAT (*Rosa rugosa*, AER58181.1), *SlAAT1* (*Solanum lycopersicum*, AFD29888.1), *CmAAT3* (*Cucumis melo*, NP_001315395.1), CmAAT4 (*Cucumis melo*, AAW51126.1), CsAAT1 (*Citrus sinensis*, ABW81204.1), CsAAT (*Camellia sinensis*, ACV74416), MiAAT (*Mangifera indica*, CAC09378), and ClAAT (*Citrus limon*, CAC09049).

In this study, a phylogenetic tree was constructed using 21 identified protein sequences from other species and 90 PmBAHDs (Fig 2). The phylogenetic tree was divided into five branches: the I branch contained 45 genes from *P*. *mume*, which corresponded to *PmBEATs*; the II branch contained 21 genes; the III branch contained 4 genes from *P*. *mume*; and the IV and V branches contained 10 genes.

The PmBAHDs in cluster I were all close to CbBEAT from *Clarkia breweri*, which was reported to be involved in benzyl acetate biosynthesis [21]. The first branch also contained four alcohol acyltransferases (AAT). CmAAT4, SAAT and RhAAT1 were involved in the synthesis of volatile esters in melon fruit, ripening strawberry fruit and rose flowers, respectively [22–24]. RrAAT was involved in monoterpene synthesis in rose flowers [25]. There were no proteins from other species with clarified functions in cluster II. The third branch contained four members. PmBAHD70 was close to PhCFAT from petunia, which used coniferyl alcohol as a substrate to form coniferyl acetate by acyl transfer [9]. PmBAHD67-69 clustered close to LtCAAT1 and LtCAAT2 from *Larrea tridentata*, which could also catalyse the formation of coniferyl acetate from coniferyl alcohol and acetyl-CoA [13]. The results indicated that the proteins of these four candidate members may be coniferyl acyltransferases. In the IV branch, MiAAT and ClAAT were putative alcohol acyltransferases related to the metabolism of aromatic substances in lemon and mango fruits, respectively. FaAAT2 in the V branch was associated with the synthesis of aromatic substances in strawberry fruit, and EjAAT1, MdAAT1, MdAAT2, and PAAT were involved in the synthesis of volatile esters in fruits [26, 27]. PmBAHD90 and alcohol acyltransferase genes from five Rosaceae plants were located together and may be involved in the metabolism of esters. In addition, PaAAT, CmAAT3 and SlAAT1 were involved in the synthesis of volatile esters in apricot, melon and tomato fruits, respectively [24, 28, 29]. In addition, it was speculated that the candidate genes of *P*. *mume* in the V branch may be related to the synthesis of volatile esters.

To further reveal the specific functions of PmBAHD proteins, conserved motifs were analysed. We performed conserved motif analyses containing not only 90 PmBAHDs from *P*. *mume* but also 21 related proteins with identified functions in other species as references. Twenty conserved motifs were identified, and the types of domains categorized in each group showed diversity. The PmBAHD proteins in the same cluster had similar motif types, which were specific to each cluster. Motif 02 contained the HxxxD domain, and motif 03 contained the DFGWG domain. As shown in Fig 2, all amino acid sequences included the characteristic domains, HxxxD and DFGWG. Motif 01, motif 06, motif 19, motif 02, motif 09, motif 05, motif 08, motif 03, and motif 18 appeared in all PmBAHDs. Additionally, some motifs were widely distributed but did not exist in certain groups, for example, groups II, III, IV and V all contained motifs 13 and 15, which were not present in group I. Motifs 14, 20, 10, 17 and 07 were present in only cluster I. Motif 11 was not present in cluster III but was present in the remaining clusters. Combining the motif analysis results with the phylogenetic analysis suggests that, genes with the same motifs may have similar functions.

### Expression pattern analysis of the potential *PmBAHDs* by transcriptome data

**Fig 3:**
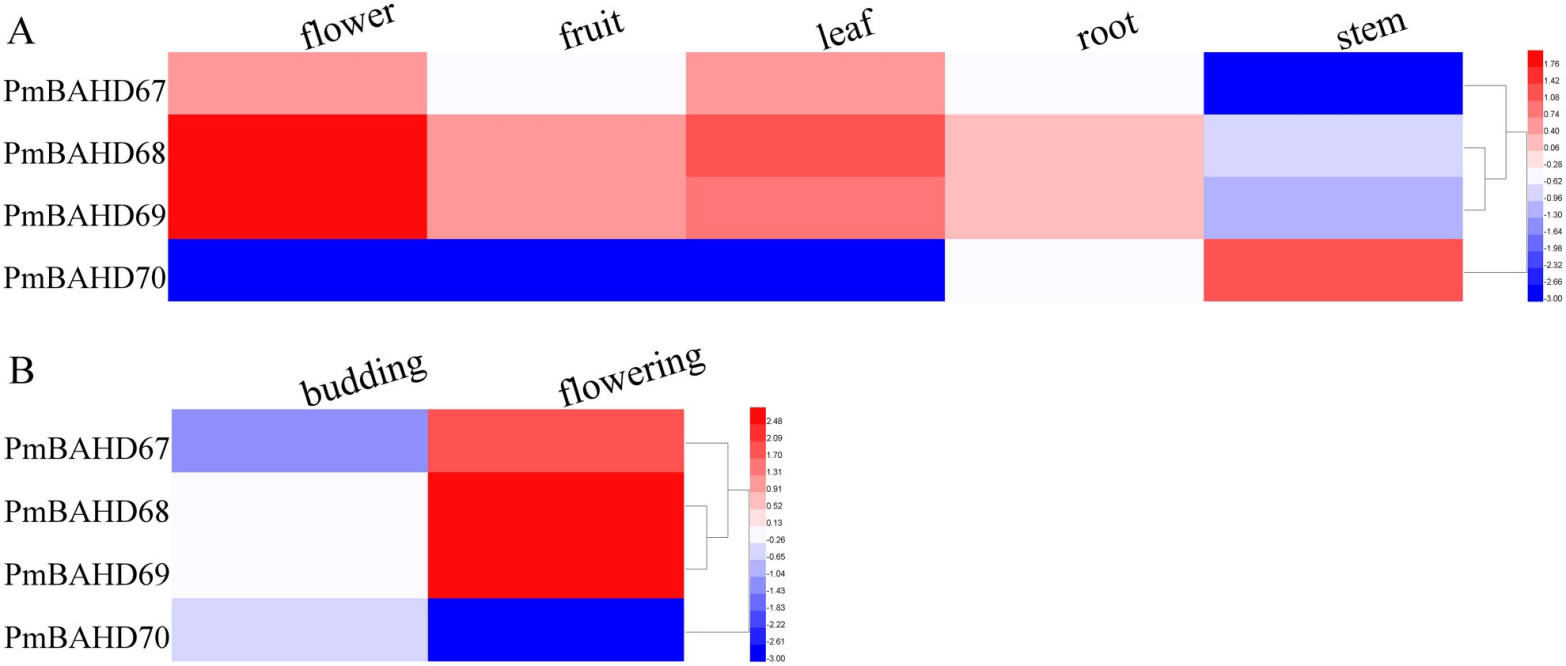
Heatmap clustering of expression patterns of *PmBAHD67-70*. (A) Expression patterns of *PmBAHD67-70* in five tissues (SRP014885) and (B) during the budding and flowering stages in transcriptome data sets (PRJCA000274). The RPKM values were log10 transformed.

The flower is the main organ emitting floral volatiles. To explore the expression profiles of candidate *PmBAHDs*, we analysed RNA-seq from flowers at developmental stages (budding stage and flowering stage) and different tissues. As shown in Fig 3, *PmBAHD67-70* genes were expressed at various levels. Among the different tissues, *PmBAHD67-69* were expressed at higher levels in flowers than in other tissues, whereas the transcript level of *PmBAHD70* was highest in stems (Fig 3A). During the developmental stages, the expression levels of *PmBAHD67-69* increased from the budding stage to the flowering stage, at which point these genes were highly expressed. However, the expression level of *PmBAHD70* decreased (Fig 3B). These results showed that *PmBAHD67-69* may be involved in floral scent metabolism.

### Isolation and sequence analysis of *PmCFATs*

**Fig 4:**
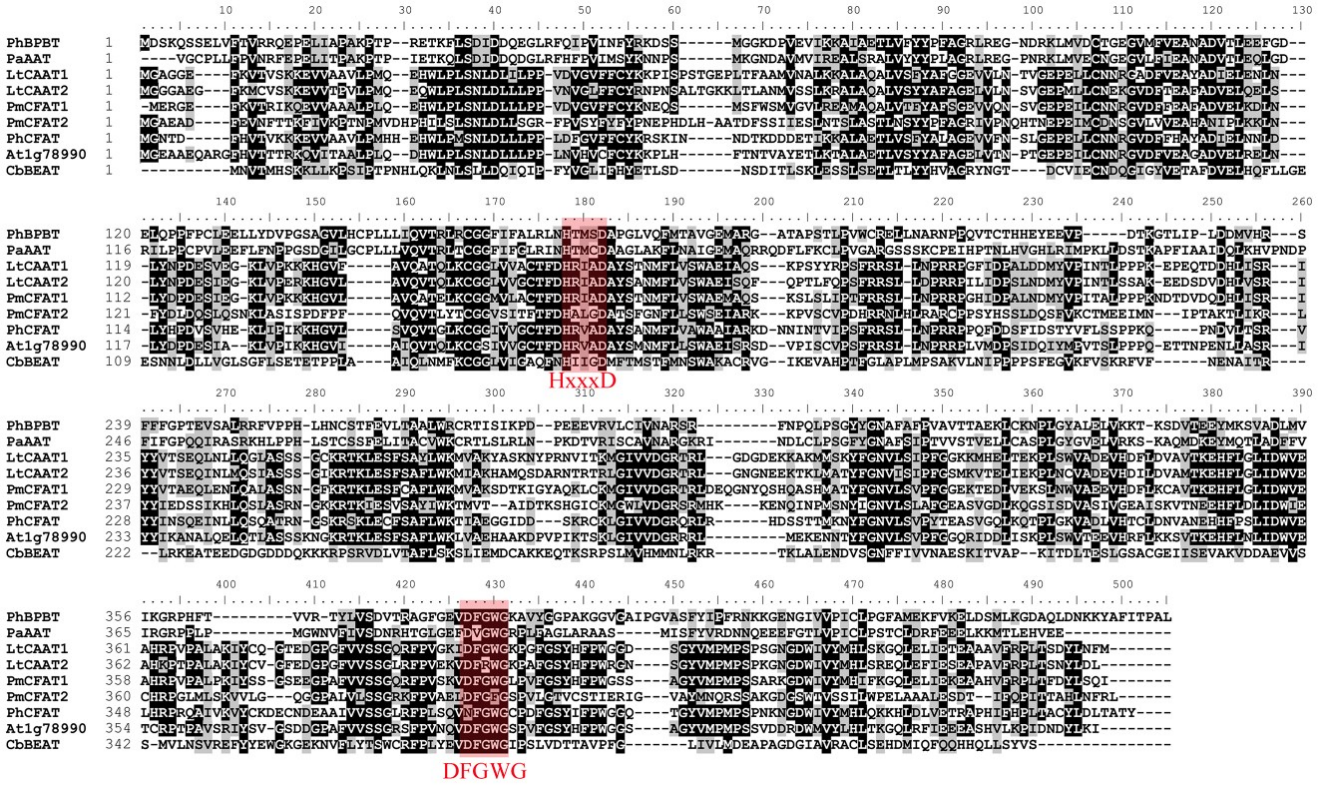
Alignment of multiple PmCFATs and related proteins. The characteristic conserved domains are framed with a red box.

Based on the results of bioinformatics analyses, the *PmBAHD67-70* genes clustered in group III were considered homologs of *PmCFAT* in *P*. *mume*. Because of the high sequence similarity of *PmBAHD67-69* (S1 and S2 Figs), one pair of primers, *PmCFATIF* and *PmCFATIR*, was designed to amplify all three cDNA sequences; however, only one sequence was obtained. The *PmCFATIIF* and *PmCFATIIR* primers were used to amplify the coding sequence of *PmBAHD70*. Finally, two cDNA sequences of *CFAT*-like genes, named *PmCFAT1* and *PmCFAT2*, were isolated from *P*. *mume* ‘Sanlunyudie’. The two cDNA sequences were 1383 bp and encoded 460 amino acids, and their predicted molecular weights were 51.61 kDa and 50.92 kDa, respectively. The sequence of *PmCFAT1* was similar to those of *PmBAHD67-69* (S3 and S4 Figs), and *PmCFAT2* was similar to *PmBAHD70* (S5 and S6 Figs) in both nucleotide and amino acid sequences. The PmCFAT1 and PmCFAT2 protein sequences contained the conserved motifs HxxxD and DFGWG (Fig 4), which are considered characteristic of the BAHD superfamily. The motif HxxxD in PmCFAT1 was more conserved than that in PmCFAT2.

### Expression patterns of *PmCFAT1* and *PmCFAT2*

**Fig 5:**
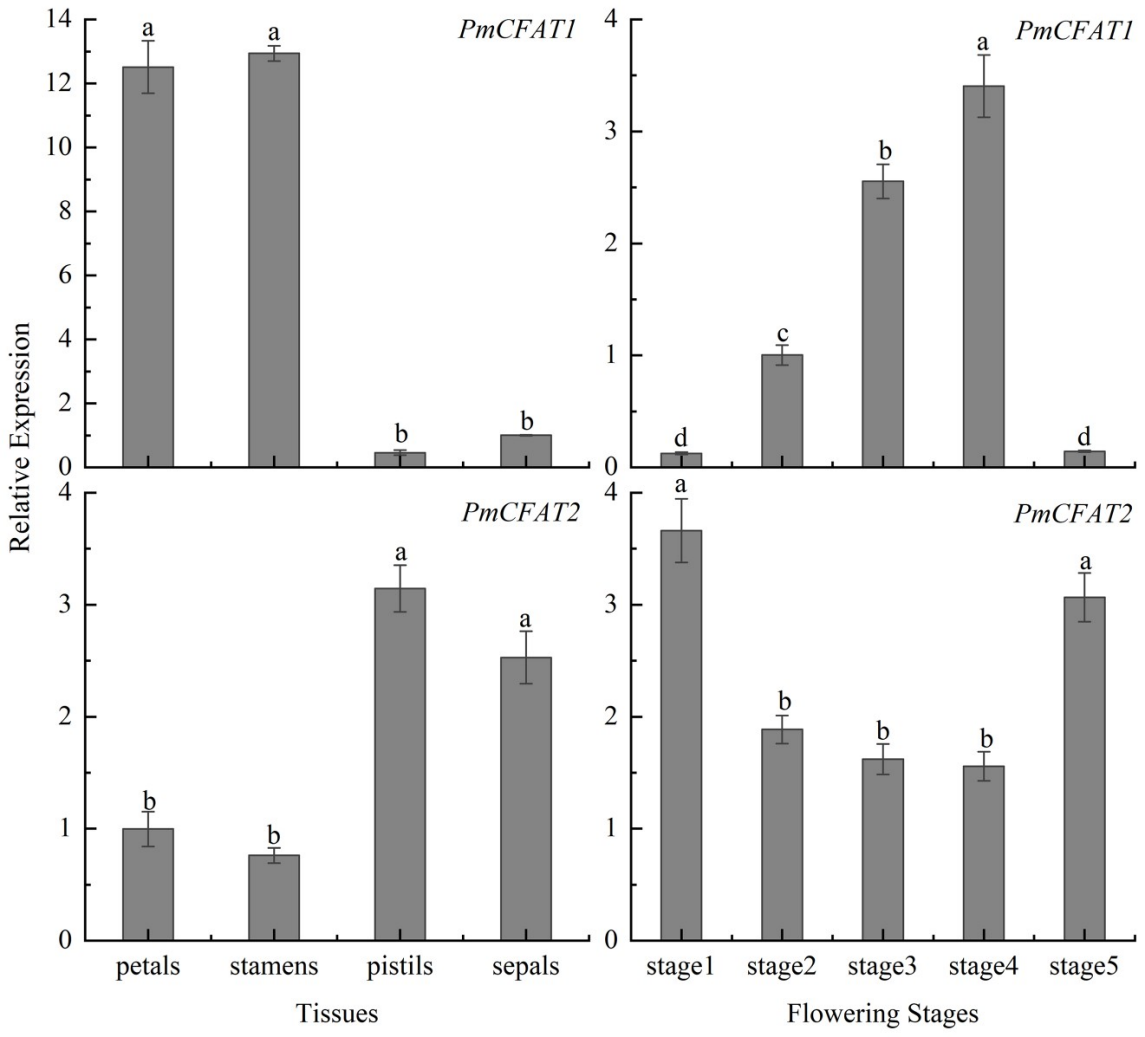
Relative expression levels of the *PmCFAT1* and *PmCFAT2* genes. (A) (C) Expression levels of *PmCFAT1* and *PmCFAT2* in different flower organs. (B) (D) Expression levels of *PmCFAT1* and *PmCFAT2* during different flowering stages.

To further analyse the possible functions of the *PmCFATs*, we used quantitative real-time (qRT)-PCR to determine the relative expression levels of *PmCFAT1* and *PmCFAT2*. *PmCFAT1* expression was higher in stamens and petals than in sepals and pistils (Fig 5A), whereas *PmCFAT2* expression was higher in pistils and sepals than in stamens and petals (Fig 5B). In addition, the expression levels of these two genes changed during flower development. The relative expression of *PmCFAT1* increased from stage 1 to stage 4, reaching a maximum level at stage 4, then declined in stage 5 (Fig 5C), which was the same progression indicated for *PmBAHD67-69* in the transcriptome data sets from the budding stage and flowering stage (Fig 3B). The expression of *PmCFAT2*, as a whole, showed the opposite tendency to that of *PmCFAT1*; it decreased from stage 1 to stage 4 and then increased in stage 5 (Fig 5D), similar to the expression of *PmBAHD70* (Fig 3B). The transcript levels of *PmCFAT1* were much higher than those of *PmCFAT2* (S7 Fig). In general, the peak in the expression pattern of *PmCFAT1* was earlier than the peaks of eugenol synthesis and release, suggesting that the gene showed a strong correlation with the release of eugenol.

### Subcellular localizations of PmCFAT1 and PmCFAT2

**Fig 6:**
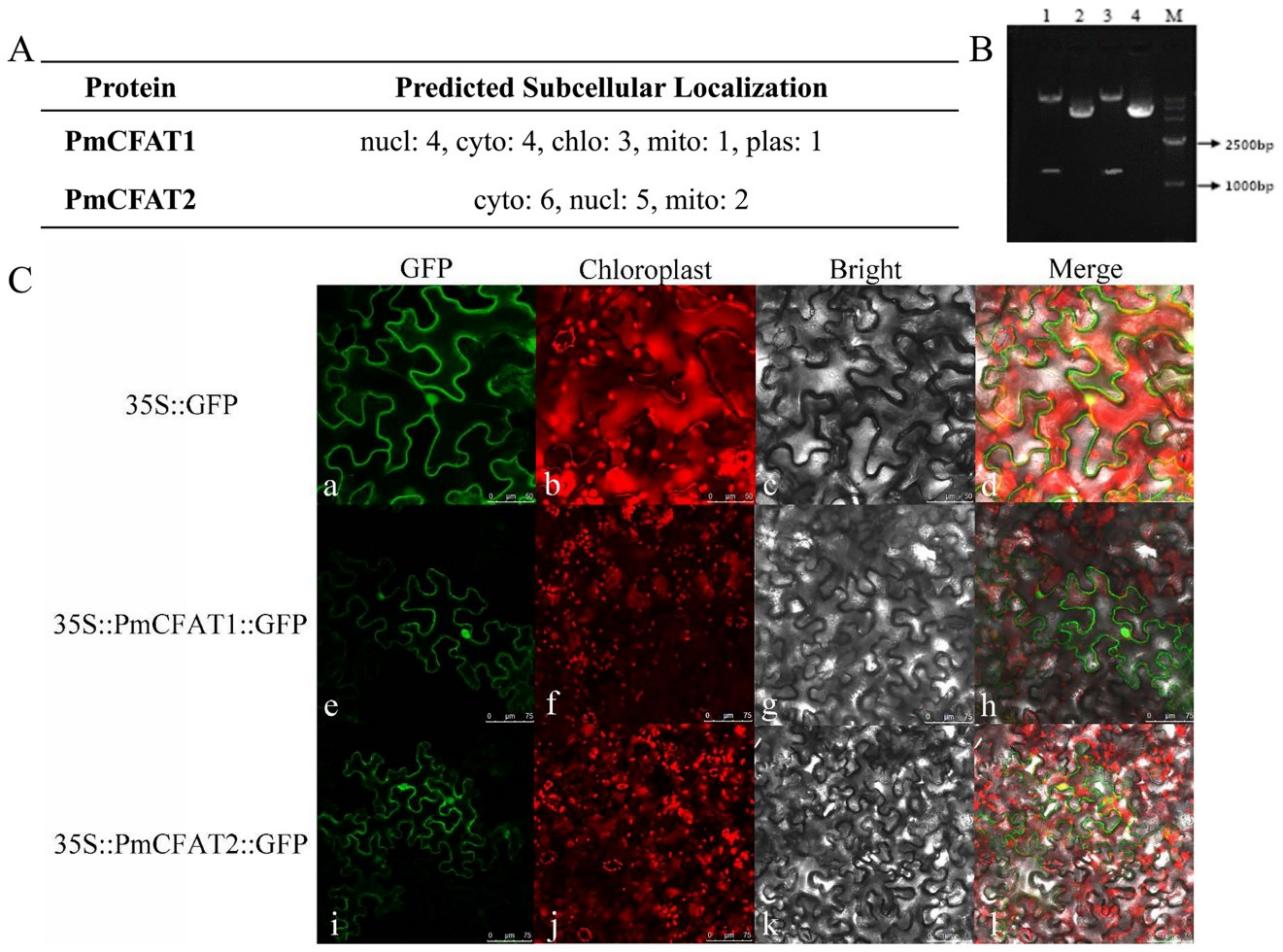
Subcellular localization analyses of PmCFAT1 and PmCFAT2. (A) Prediction of protein subcellular localization of PmCFATs with WoLF PSORT Server. Note: nucl represents nucleus; cyto represents cytoplasm; mito represents mitochondria; plas represents plasma membrane; chlo represents chloroplast. (B) Restriction enzyme digestion of *pSuper1300-GFP-PmCFATs*. M: DL 15,000 Marker; 1 and 3 represent the enzyme digestion products of *pSuper1300*::*PmCFAT1*::*GFP* and *pSuper1300*::*PmCFAT2*::*GFP*, respectively; 2 and 4 represent circular plasmids of *pSuper1300*::*PmCFAT1*::*GFP* and *pSuper1300*::*PmCFAT2*::*GFP*, respectively, as controls. (C) Subcellular localization of PmCFAT1 and PmCFAT2. The fusion proteins were observed under a confocal laser scanning microscope. a, e, i show the green fluorescence channel; b, f, j show the chloroplast autofluorescence channel. c, g and k show the bright field channel; d, h and l were created from the images shown in the first two panels. a-d: bar = 10 μm; e-l: bar = 15 μm.

The prediction of protein subcellular localization with the WoLF PSORT Server showed that PmCFAT1 was located in the cytoplasm and nucleus with a probability of 30.77%, and PmCFAT2 was located in the cytoplasm with a probability of 46.15% and in the nucleus with a probability of 38.46% (Fig 6A). To investigate the intracellular localization of these two proteins, ORF sequences with restriction enzyme cutting sites were obtained and ligated into the digested *pSuper*::*1300*::*GFP* vector by T4 ligase. Then, double digestion with a restriction enzyme showed that the recombinant expression vectors derived from the CaMV-35S promoter were successfully constructed (Fig 6B). Transient transformation of tobacco leaves was used to examine protein localization with the empty vector as a control. At 48–56 h after injection, the tobacco leaves were collected to observe the green fluorescence. The results showed that the proteins encoded by PmCFAT1 and PmCFAT2 were located in the cytoplasm and nucleus (Fig 6C).

### Functional characterization of PmCFAT genes in vitro

**Fig 7:**
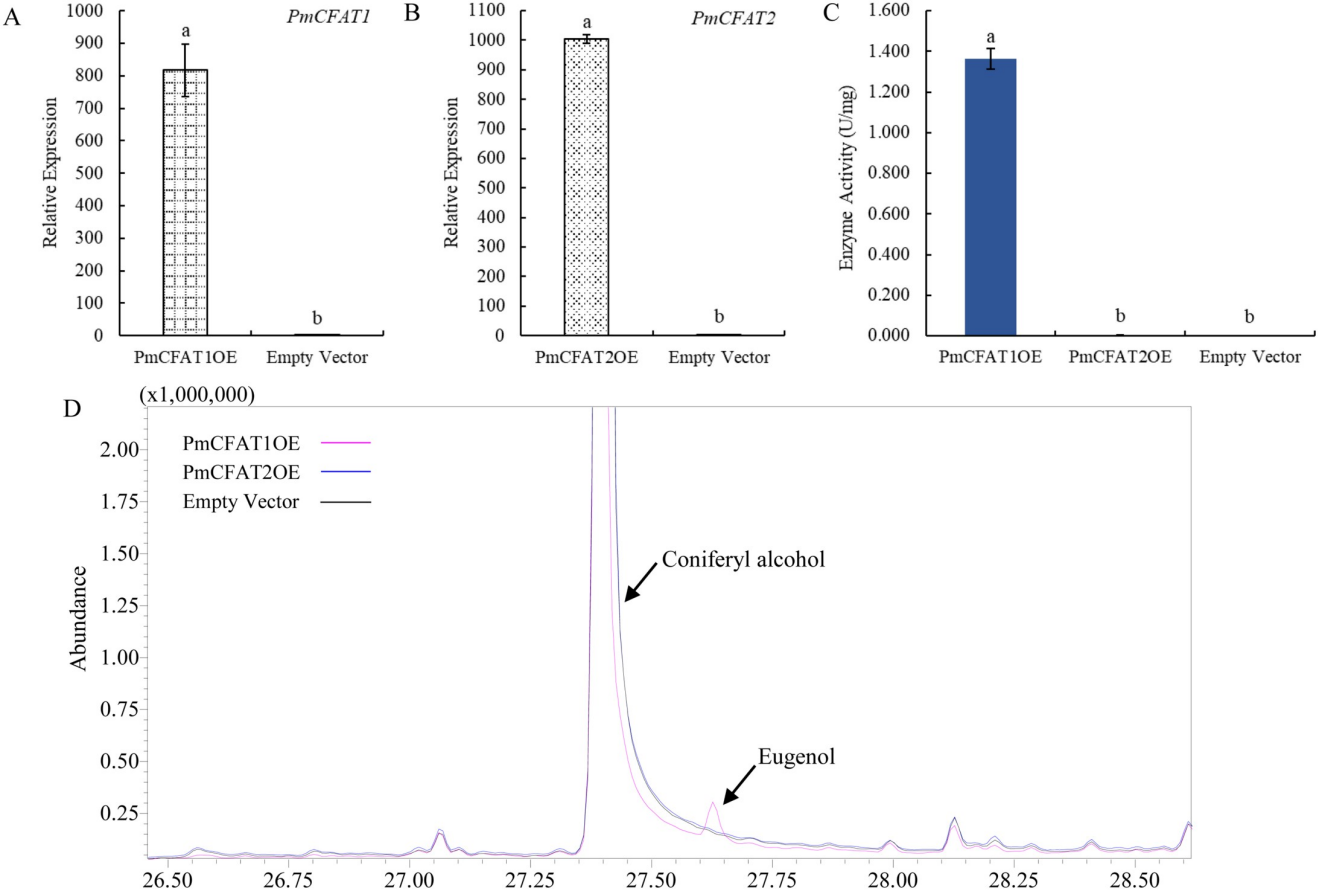
Enzyme activity analyses of PmCFAT1 and PmCFAT2. (A) and (B) Expression levels of *PmCFAT1* and *PmCFAT2* in tobacco leaves transformed with these *PmCFAT* genes, respectively. (C) Coniferyl alcohol acetyltransferase activities of PmCFAT1 and PmCFAT2. (D) Detection of eugenol in the enzyme activity assays by GC-MS.

To further explain the potential roles of the candidate *PmCFAT* genes in eugenol biosynthesis, they were transiently expressed in the leaves of tobacco (*N*. *benthamiana*). The gene expression levels in tobacco leaves were detected using qRT-PCR (Fig 7A and 7B). The expression levels of *PmCFAT1* and *PmCFAT2* in transgenic leaves were far higher than those in the control (empty vector). Then, as shown in Fig 7C, obvious enzyme activities were detected in the leaves, overexpressing *PmCFAT1*, compared with those overexpressing *PmCFAT2* and those transformed with the empty vector. Meanwhile, eugenol was produced through an enzyme assay with protein extracts overexpressing *PmCFAT1*, whereas none was detected with the *PmCFAT2* and empty vector extracts (Fig 7D). The results indicated that PmCFAT1, but not PmCFAT2, may be involved in eugenol synthesis in *P*. *mume*.

## Discussion

Floral scents, which are composed of a diversity of compounds with low molecular weights, are secondary metabolites mainly derived from terpenoids, phenylpropanoids/benzenoids and fatty acid derivatives [30]. Phenylpropanoids such as eugenol and isoeugenol contribute substantially to the flavours of fruits and the scents of floral plants [31]. *P*. *mume* is an important ornamental tree in Southern China, blooming in early spring with a pleasant floral fragrance [5]. When hybrids of *P*. *mume* and other *Prunus* species are bred, they possess a strong capacity for cold resistance when introduced into Northern China; however, these hybrids lose the characteristic scent of *P*. *mume* [5]. To date, volatile organic compounds from *P*. *mume* with fragrance have been identified. Previous studies on *Prunus* species have shown that eugenol existed in only the scented species (*P*. *mume*) and cultivars and was not detected in non-scented species, even in trace amounts [6, 14]. *P*. *mume* ‘Sanlunyudie’, with a typical fragrance due to a specific mixture of benzyl acetate and eugenol, was selected as a plant material to elucidate the molecular mechanism of eugenol production.

The emission quantities of floral fragrance exhibit spatial and temporal rhythms. Of course, flowers releasing floral volatiles provide the plant material for most studies about floral scents. For instance, the volatile compounds identified in *Freesia* flowers are mainly terpenes and carotenoid derivatives [32]. Furthermore, petals are the main organs that release distinct aromatic compounds in many plants [25]. In lily, the amounts of floral volatiles were the highest in petals [33], and a study in roses showed that the volatile components and their amounts in the whole flower were generally the same as those in the petals [34]. However, in *Camellia buxifolia*, the volatile compound abundance and number in stamens were greater than in petals [35]. In our present study, eugenol was emitted mainly from the petals and stamens of *P*. *mume* ‘Sanlunyudie’.

The emission amounts of floral volatiles often increase with flower blossoming and decrease with withering. Basically, the release of volatiles first increased and then decreased. Benzyl acetate was the main compound in *P*. *mume*. From the budding stage to the full bloom stage, the emission amounts were increased, and then, they were reduced in the fading stage [36]. Similarly, all volatile compounds presented similar trends during flower development in *C*. *buxifolia*: initially increased from bud to half-opening, at which maximum emission was reached, and then decreased in complete opening and fall [35]. Unlike the release profile of benzyl acetate in flowers, eugenol emission abundance rose from budding through the fading stage and peaked in the fading stage in *P*. *mume*. In the end, eugenol is the major component of the floral scent at the flower withering stage.

CFAT, a key enzyme that converts a pool of substrate to phenylpropanoids [7], may play an important role in the synthesis of eugenol from mei. To obtain functional CFAT, in the present study, 90 candidate *BAHD* genes were screened based on the genome database of mei. CFATs all belong to the BAHD acyltransferase family because they contain HxxxD and DFGWG conserved domains, which reflects, to some extent, the presence of a large number of BAHD family genes in mei. Candidate PmBAHDs were divided into five major branches of the phylogenetic tree based on having a phylogenetic cluster pattern that is similar to that of BAHD family members in *Populus* and *Arabidopsis* [20]. Due to the different classification standards and analysis software used, the classifications for the evolutionary relationships within the BAHD family differed. In a classical example of an evolution-based classification, 46 BAHD acyltransferases were identified by D’uria. The 46 members were evolutionarily divided into five major branches, and the substrate types and enzyme activities of each branched protein were different. Most of the members in branch I function as modifiers of phenol glycosides. The members in branch II were related to the prolongation of stratum corneum wax and defence against pathogens. The members of branch III were mainly alcohol acyltransferases, which participated in the synthesis of volatile esters. Branch IV featured only one agmatine coumaroyltransferase (ACT), and branch V was divided into three subfamilies, one of which was related to the synthesis of volatile esters [37]. Although the phylogenetic tree was divided into eight clades, consisting of 69 BAHD genes from *Arabidopsis*, *Populus*, *Medicago*, *Vitis* and *Oryza*, the classifications were basically similar to those of D’uria [38]. There were some differences in the member distribution, subgroup evolution and branching of specific individual genes in each branch of the phylogenetic tree we generated, which showed that the candidate genes may not be all homologous genes of *CFAT* and may include other types of BAHD family members. The proteins in different clusters exhibited some selectivity for substrates and acyl donors [39, 40]. For example, the 45 PmBAHDs of the first branch were closely related to CbBEAT, which may catalytic benzyl alcohol as a substrate [21]. Similarly, three members with benzyl alcohol acetyltransferase activity have been proven. Among these three, the overexpressed of two members enhanced benzyl acetate production in the petal protoplasts of *P*. *mume* [15]. In the third branch, PhCFAT, LtCAAT1 and LtCAAT2 were associated with acetyl-CoA as the acyl donor. PhCFAT catalysed the activity of coniferyl alcohol at pH = 6.0 and showed strong substrate selectivity [9]. The optimal substrate for LtCAAT1 and LtCAAT2 was coniferyl alcohol, which could also catalyse coumarinol and caffeol orsinal alcohol [13]. It is speculated that the proteins encoded by the four candidate genes of *P*. *mume* in the third branch may have catalytic activity on coniferyl alcohol. The V family gene has broad selectivity for acyl groups, such as acetyl [41] and benzoyl [42, 43] donors and various medium chain fats. An acyl donor [26] is a donor of an acyl group. The motif composition in every cluster was characterized by the same or similar structure [44], suggesting functional similarities among the CFAT proteins within the same group. For example, CFAT proteins, predicted to be alcohol acyltransferases, clustered in group I with those containing the same conserved motifs. Similar results appeared in the analysis of conserved motifs in FLA proteins of *Populus*. Different groups contained distinct conserved motifs; for example, motifs 1 and 2 appeared in nearly all members of group A. Motifs 5, 6, 7, 9, 12, 13 were conserved in group B. Motifs 8, 10, 11, 19 were specific to group C. Motifs 18 and 20 existed only in group D [44]. Finally, the *PmBAHD67-70* genes in group three may be CFAT-like genes. *PmCFAT1* and *PmCFAT2* were isolated from *P*. *mume* ‘Sanlunyudie’ using the CDSs of *PmBAHD67-70*.

The biosynthesis of volatile compounds in plants is usually regulated at the transcriptional level [45]. The expression levels of genes contributing to the monoterpene pathway were higher in petals than in other organs of *Lilium* [33]. The expression level of *PmCFAT1* was higher in petals and stamens than in the remaining organs, consistent with the pattern of eugenol release. Similar expression patterns of *CFAT* genes were also observed in other plants. For instance, *PhCFAT* in petunia has its highest expression in petals but is not expressed in other flower parts or the roots, stems or leaves [9]. Genes involved in biosynthesis were expressed during plant development. The transcription of *PmBAHD* genes related to benzyl acetate was enhanced from F1 to F3 and then decreased through F4 [15]. The expression level of *PhCFAT* identified in the petunia hybrid was low in the bud stage and the initial stage of corolla development, was highest when the flower was in complete full bloom, and then decreased after 2 days of blooming. The regular pattern of *PhCFAT* was consistent with the emission of eugenol from petunia [9]. Although *PmCFAT1* was initially expressed at a low level at the budding stage and was apparently increased at the full bloom stage, it was subsequently decreased at the wilting stage, while the synthesis and emission quantity of eugenol increased from the budding stage to the wilting stage. During the flowering process, there was a specific time difference between the peak expression of the genes encoding the floral scent biosynthetic proteins and the emission quantities of the floral components, and sometimes this difference was as much as 1–2 days, which also indicated that the expression of the floral-scent-related genes ultimately affects floral volatile compound synthesis [46, 47]. Therefore, *PmCFAT1* may play the main role in eugenol synthesis in *P*. *mume*.

Coniferyl alcohol is the precursor of eugenol. CFAT catalyses coniferyl alcohol to coniferyl acetate, which can be converted to eugenol by EGS. It has been reported that the amount of eugenol was significantly increased in transgenic aspen leaves, when *PhCFAT* was transferred into hybrid aspen. However, when neither simultaneous transfer of *PhCFAT* and *PhEGS* nor individual transfer of *PhEGS* occurred, the yield of eugenol in transgenic plants was only slightly increased and was lower than that in plants with *PhCFAT* alone. Thus, CFAT is a rate-limiting enzyme in the biosynthetic pathway of eugenol [13]. However, overexpression of PhCFAT in transgenic tobacco did not cause any synthesis of eugenol in Koeduka et al.’s work. We hypothesized that this may be caused by a lack of coniferyl alcohol in tobacco leaves. In our study, eugenol was produced in an in vitro enzyme activity assay using the crude protein of tobacco leaves overexpressing PmCFAT1, although the intermediate coniferyl acetate was not detected. This result indicated that the reaction from coniferyl acetate to eugenol by EGS took place in the reaction system in vitro, which means that EGS activity exists in tobacco leaves.

Overall, in this study, we found temporal and spatial changes in the release of eugenol. Four *CFAT-like* genes, which may be related to eugenol biosynthesis in *P*. *mume*, were screened according to genome database and phylogenetic analyses. Using these 4 gene sequences, we cloned 2 *PmCFAT* genes from *P*. *mume* ‘Sanlunyudie’, which has a typical pleasant floral scent. Bioinformatics analysis and expression profiles showed that *PmCFAT1*, rather than *PmCFAT2*, may play an important role in eugenol biosynthesis. This study provided some evidence for the biological function of *PmCFAT1*; however, this topic needs further study. Meanwhile, in vitro functional characterization indicated that *PmCFAT1* contributed to the synthesis of eugenol.

## Supporting information

S1 Table Inventory and characteristics of the *PmBAHD* genes identified in *P*. *mume*. (DOCX)

S1 Fig cDNA sequence alignment of *PmBAHD67-69* from the *P*. *mume* genome. (TIF)

S2 Fig Protein sequence alignment of PmBAHD67-69 from the *P*. *mume* genome. (TIF)

S3 Fig cDNA sequence alignment of *PmBAHD67-69* and *PmCFAT1* isolated from *P*. *mume*. (TIF)

S4 Fig Protein sequence alignment of PmBAHD67-69 and PmCFAT1 isolated from *P*. *mume*. (TIF)

S5 Fig cDNA sequence alignment of *PmBAHD70* and *PmCFAT2* isolated from *P*. *mume*. (TIF)

S6 Fig Protein sequence alignment of PmBAHD70 and PmCFAT2 isolated from *P*. *mume*. (TIF)

S7 Fig Cq values of *PmCFAT1* and *PmCFAT2* in different organs and during flower development stages of *P*. *mume*. (TIF)
