# Supplementary material for: Genome-wide identification, characterization, expression and enzyme activity analysis of coniferyl alcohol acetyltransferase genes involved in eugenol biosynthesis in Prunus mume
Source: PLoS One. 2019 Oct 16;14(10):e0223974. doi: 10.1371/journal.pone.0223974 (PMC6795479; doi:10.1371/journal.pone.0223974)
Supplement: S1 Table — (DOCX) [file pone.0223974.s001.docx]

Table 1 Inventory and characteristics of the *PmBAHD* genes identified in *P. mume*.

| **Name** | **ID** | **Locus** | **Length** | **MW** | **Introns** | **DFGWG Domain** |
| --- | --- | --- | --- | --- | --- | --- |
| *PmBAHD01* | *Pm010992* | Pm3:8229556:8230902 | 448 | 49.11 | 0 | NFGWG |
| *PmBAHD02* | *Pm010994* | Pm3:8236905:8238248 | 447 | 49.14 | 0 | NFGWG |
| *PmBAHD03* | *Pm010989* | Pm3:8204439:8205755 | 438 | 48.91 | 0 | NFGWG |
| *PmBAHD04* | *Pm010988* | Pm3:8192054:8193376 | 440 | 48.66 | 0 | DFGWG |
| *PmBAHD05* | *Pm010979* | Pm3:8129028:8130350 | 440 | 48.71 | 0 | DFGWG |
| *PmBAHD06* | *Pm010980* | Pm3:8131250:8132614 | 454 | 50.23 | 0 | NFGWG |
| *PmBAHD07* | *Pm010977* | Pm3:8119387:8120736 | 449 | 49.57 | 0 | NFGWG |
| *PmBAHD08* | *Pm010990* | Pm3:8215947:8217296 | 449 | 49.74 | 0 | NFGWG |
| *PmBAHD09* | *Pm010985* | Pm3:8161540:8162847 | 435 | 48.14 | 0 | EFGWG |
| *PmBAHD10* | *Pm010984* | Pm3:8158666:8159994 | 442 | 49.06 | 0 | NFGWG |
| *PmBAHD11* | *Pm010986* | Pm3:8169726:8171039 | 437 | 48.52 | 0 | NFGWG |
| *PmBAHD12* | *Pm011005* | Pm3:8313900:8315240 | 446 | 49.26 | 0 | DFGWG |
| *PmBAHD13* | *Pm011001* | Pm3:8273507:8274847 | 446 | 49.14 | 0 | DFGWG |
| *PmBAHD14* | *Pm026103* | Pm8:7786341:7787702 | 453 | 50.13 | 0 | DFGWG |
| *PmBAHD15* | *Pm010971* | Pm3:8086964:8088316 | 450 | 50.16 | 0 | DFGWG |
| *PmBAHD16* | *Pm010998* | Pm3:8256871:8258223 | 450 | 49.97 | 0 | DFGWG |
| *PmBAHD17* | *Pm010996* | Pm3:8247708:8249048 | 446 | 49.89 | 0 | DFGWG |
| *PmBAHD18* | *Pm010975* | Pm3:8113148:8114512 | 454 | 50.55 | 0 | DFGWG |
| *PmBAHD19* | *Pm011503* | Pm3:11571549:11572982 | 477 | 53.15 | 0 | DFGWG |
| *PmBAHD20* | *Pm011010* | Pm3:8371395:8372819 | 474 | 52.47 | 0 | NFGWG |
| *PmBAHD21* | *Pm016771* | Pm5:4567054:4568481 | 475 | 52.41 | 0 | DFGWG |
| *PmBAHD22* | *Pm011011* | Pm3:8377092:8378510 | 472 | 52.23 | 0 | DFGWG |
| *PmBAHD23* | *Pm011009* | Pm3:8362016:8363443 | 475 | 52.25 | 0 | DFGWG |
| *PmBAHD24* | *Pm010976* | Pm3:8115043:8116374 | 443 | 49.25 | 0 | DFGWG |
| *PmBAHD25* | *Pm010967* | Pm3:8058923:8060353 | 476 | 52.43 | 0 | DFGWG |
| *PmBAHD26* | *Pm010974* | Pm3:8110118:8111458 | 446 | 49.04 | 0 | SFGWG |
| *PmBAHD27* | *Pm006868* | Pm2:19710735:19712075 | 446 | 49.08 | 0 | DFGWG |
| *PmBAHD28* | *Pm006864* | Pm2:19680726:19682066 | 446 | 49.34 | 0 | DFGWG |
| *PmBAHD29* | *Pm025606* | Pm8:3400815:3402209 | 464 | 51.77 | 0 | DFGWG |
| *PmBAHD30* | *Pm025612* | Pm8:3451935:3453828 | 506 | 56.09 | 1 | DFGWG |
| *PmBAHD31* | *Pm006894* | Pm2:19864620:19865939 | 439 | 49.60 | 0 | DFGWG |
| *PmBAHD32* | *Pm006849* | Pm2:19561090:19562397 | 435 | 48.83 | 0 | DFGWG |
| *PmBAHD33* | *Pm006850* | Pm2:19568381:19569688 | 435 | 49.05 | 0 | DFGWG |
| *PmBAHD34* | *Pm006848* | Pm2:19535928:19537181 | 417 | 46.61 | 0 | DFGWG |
| *PmBAHD35* | *Pm006860* | Pm2:19654008:19655336 | 442 | - | 0 | DFGWG |
| *PmBAHD36* | *Pm006862* | Pm2:19670279:19672730 | 494 | 55.69 | 1 | DFGWG |
| *PmBAHD37* | *Pm006859* | Pm2:19652034:19653344 | 436 | 48.92 | 0 | DFGWG |
| *PmBAHD38* | *Pm010968* | Pm3:8071297:8072757 | 486 | 54.47 | 0 | DFGWG |
| *PmBAHD39* | *Pm010964* | Pm3:8042550:8043602 | 350 | 39.97 | 0 | DFGWG |
| *PmBAHD40* | *Pm010973* | Pm3:8107320:8109158 | 468 | 52.92 | 2 | DFGWG |
| *PmBAHD41* | *Pm006858* | Pm2:19648658:19649992 | 444 | 49.95 | 0 | DFGWG |
| *PmBAHD42* | *Pm006854* | Pm2:19597787:19599115 | 442 | 50.13 | 0 | DFGLG |
| *PmBAHD43* | *Pm025400* | Pm8:916180:917508 | 442 | 49.79 | 0 | DFGWG |
| *PmBAHD44* | *Pm006869* | Pm2:19718018:19719346 | 442 | 49.77 | 0 | DFGWG |
| *PmBAHD45* | *Pm006857* | Pm2:19640968:19642296 | 442 | 49.96 | 0 | DFGWG |
| *PmBAHD46* | *Pm016035* | Pm4:22785643:22786857 | 404 | 44.61 | 0 | DFGWG |
| *PmBAHD47* | *Pm016036* | Pm4:22790088:22791476 | 462 | 50.86 | 0 | DFGWG |
| *PmBAHD48* | *Pm016033* | Pm4:22780252:22781655 | 467 | 51.43 | 0 | DFGWG |
| *PmBAHD49* | *Pm016034* | Pm4:22782227:22783684 | 485 | 54.40 | 0 | DFGWG |
| *PmBAHD50* | *Pm016047* | Pm4:22837545:22838963 | 472 | 52.83 | 0 | DFGWG |
| *PmBAHD51* | *Pm016046* | Pm4:22835282:22836649 | 455 | 51.22 | 0 | DFGWG |
| *PmBAHD52* | *Pm016048* | Pm4:22841552:22842955 | 467 | 51.10 | 0 | DFGWG |
| *PmBAHD53* | *Pm016037* | Pm4:22794486:22800032 | 664 | 73.27 | 4 | DFGWG |
| *PmBAHD54* | *Pm016042* | Pm4:22818838:22820250 | 470 | 51.68 | 0 | DFGWG |
| *PmBAHD55* | *Pm016045* | Pm4:22833351:22834766 | 471 | 51.81 | 0 | DFGWG |
| *PmBAHD56* | *Pm019293* | Pm5:23165519:23167351 | 444 | 49.17 | 1 | DFGWG |
| *PmBAHD57* | *Pm026403* | Pm8:9655576:9656928 | 450 | 49.93 | 0 | DFGWG |
| *PmBAHD58* | *Pm026402* | Pm8:9650495:9651835 | 446 | 49.36 | 0 | DFGWG |
| *PmBAHD59* | *Pm026398* | Pm8:9638965:9640307 | 414 | 45.73 | 1 | DFGWG |
| *PmBAHD60* | *Pm026397* | Pm8:9636899:9638228 | 417 | 46.78 | 1 | DFGWG |
| *PmBAHD61* | *Pm026406* | Pm8:9672738:9674075 | 445 | 49.51 | 0 | DFGWG |
| *PmBAHD62* | *Pm002535* | Pm1:20112912:20114222 | 436 | 48.18 | 0 | DFGWG |
| *PmBAHD63* | *Pm018886* | Pm5:21046654:21048147 | 497 | 54.89 | 0 | DFGWG |
| *PmBAHD64* | *Pm005307* | Pm2:10329442:10330928 | 441 | 49.36 | 2 | EFGMG |
| *PmBAHD65* | *Pm005308* | Pm2:10336484:10337962 | 442 | 49.78 | 1 | QFGMG |
| *PmBAHD66* | *Pm005309* | Pm4:22785643:22786857 | 500 | 56.05 | 0 | QFGMG |
| *PmBAHD67* | *Pm030674* | scaffold590:111496:113065 | 460 | 52.07 | 1 | DFRWG |
| *PmBAHD68* | *Pm030672* | scaffold590:106295:107681 | 341 | 38.35 | 2 | DFGWG |
| *PmBAHD69* | *Pm013138* | Pm4:2016972:2018549 | 458 | 51.53 | 1 | DFGWG |
| *PmBAHD70* | *Pm023001* | Pm7:1212296:1213678 | 460 | 51.00 | 0 | DFGFG |
| *PmBAHD71* | *Pm013820* | Pm4:6963841:6966645 | 440 | 48.71 | 1 | DFGWG |
| *PmBAHD72* | *Pm013822* | Pm4:6976468:6979576 | 440 | 48.75 | 1 | DFGWG |
| *PmBAHD73* | *Pm013827* | Pm4:7060756:7062514 | 406 | 45.01 | 1 | DFGWG |
| *PmBAHD74* | *Pm013823* | Pm4:7011573:7013207 | 401 | 43.83 | 1 | DFGWG |
| *PmBAHD75* | *Pm013834* | Pm4:7120453:7123051 | 438 | 48.49 | 1 | DFGWG |
| *PmBAHD76* | *Pm013831* | Pm4:7085224:7086087 | 287 | 32.25 | 0 | DFGWG |
| *PmBAHD77* | *Pm016482* | Pm5:1891010:1892374 | 454 | 51.85 | 0 | DFGWG |
| *PmBAHD78* | *Pm016484* | Pm5:1899575:1900939 | 454 | 51.06 | 0 | DFGWG |
| *PmBAHD79* | *Pm008812* | Pm2:36359143:36360531 | 462 | 51.50 | 0 | DFGWG |
| *PmBAHD80* | *Pm008809* | Pm2:36340672:36342036 | 454 | 50.40 | 0 | DFGWG |
| *PmBAHD81* | *Pm010122* | Pm3:2665804:2667497 | 468 | - | 1 | DFGWG |
| *PmBAHD82* | *Pm024545* | Pm7:12941879:12943441 | 432 | 48.03 | 1 | DFGWG |
| *PmBAHD83* | *Pm016126* | Pm4:23262188:23263778 | 446 | 49.21 | 1 | DFGWG |
| *PmBAHD84* | *Pm027450* | Pm8:15293615:15295006 | 463 | 52.04 | 0 | DFGWG |
| *PmBAHD85* | *Pm018954* | Pm5:21421141:21422526 | 461 | 50.34 | 0 | DFGMG |
| *PmBAHD86* | *Pm005867* | Pm2:13415502:13418503 | 475 | 53.61 | 1 | DFGEG |
| *PmBAHD87* | *Pm014352* | Pm4:11958460:11960472 | 470 | 51.96 | 1 | DFGWG |
| *PmBAHD88* | *Pm017753* | Pm5:13849554:13851826 | 461 | 51.27 | 1 | DFGWG |
| *PmBAHD89* | *Pm023029* | Pm7:1452698:1456070 | 447 | 49.98 | 1 | DVGWG |
| *PmBAHD90* | *Pm023027* | Pm7:1422115:1425627 | 460 | 51.08 | 1 | SFGWG |
