# Supplementary figures and images for: Genome-wide identification, characterization, expression and enzyme activity analysis of coniferyl alcohol acetyltransferase genes involved in eugenol biosynthesis in Prunus mume
Source: PLoS One. 2019 Oct 16;14(10):e0223974. doi: 10.1371/journal.pone.0223974 (PMC6795479; doi:10.1371/journal.pone.0223974)

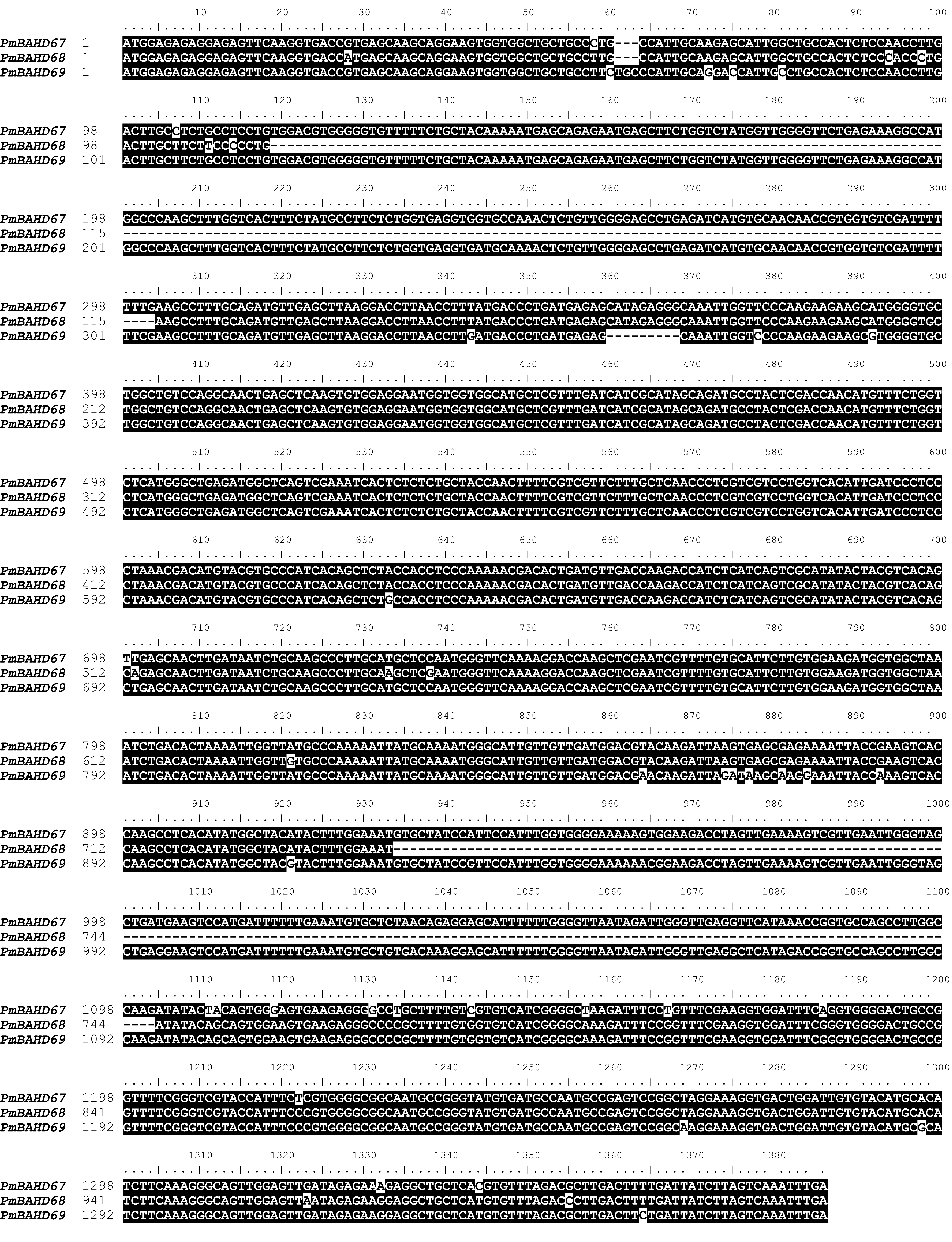

Supplement: S1 Fig — (TIF) [file pone.0223974.s002.tif]

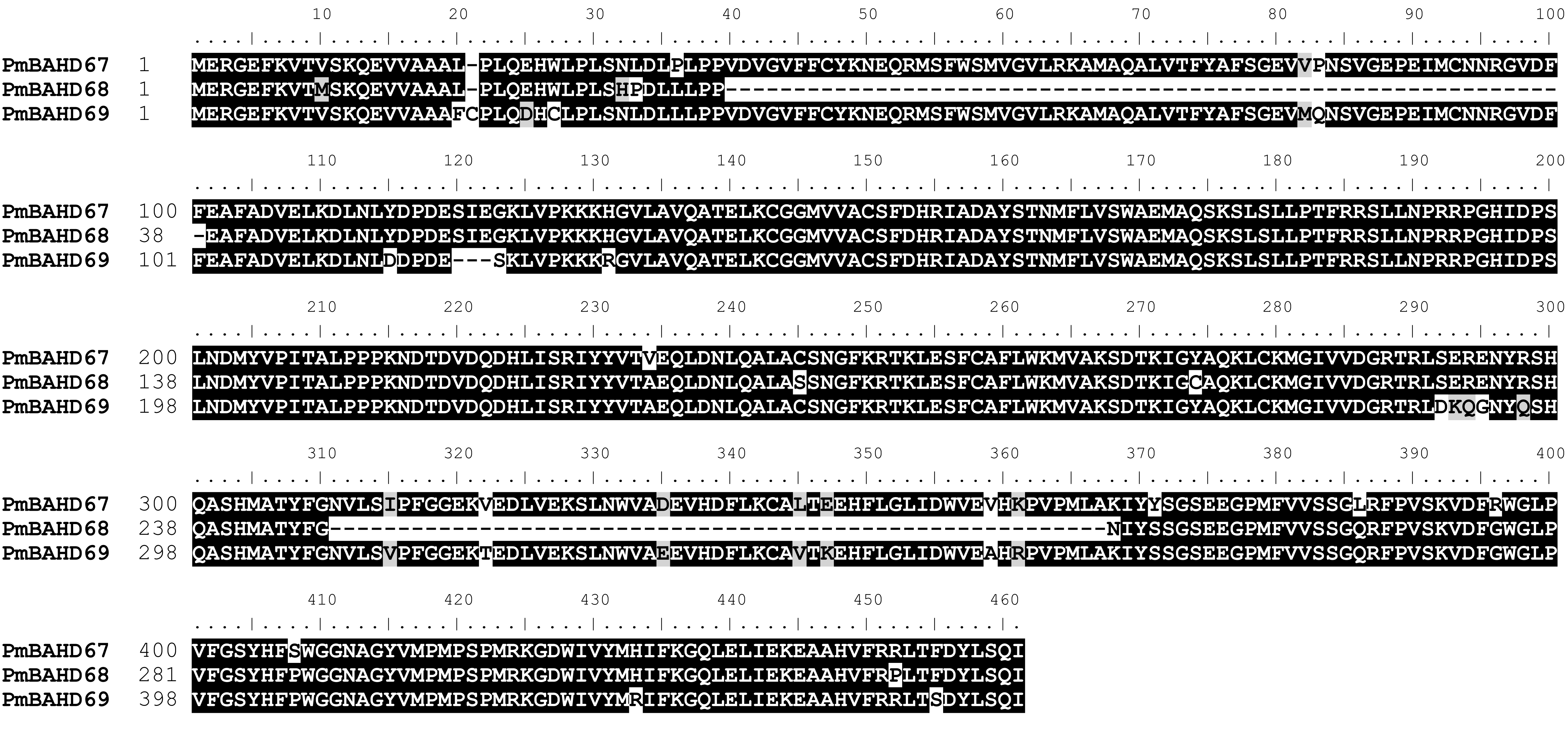

Supplement: S2 Fig — (TIF) [file pone.0223974.s003.tif]

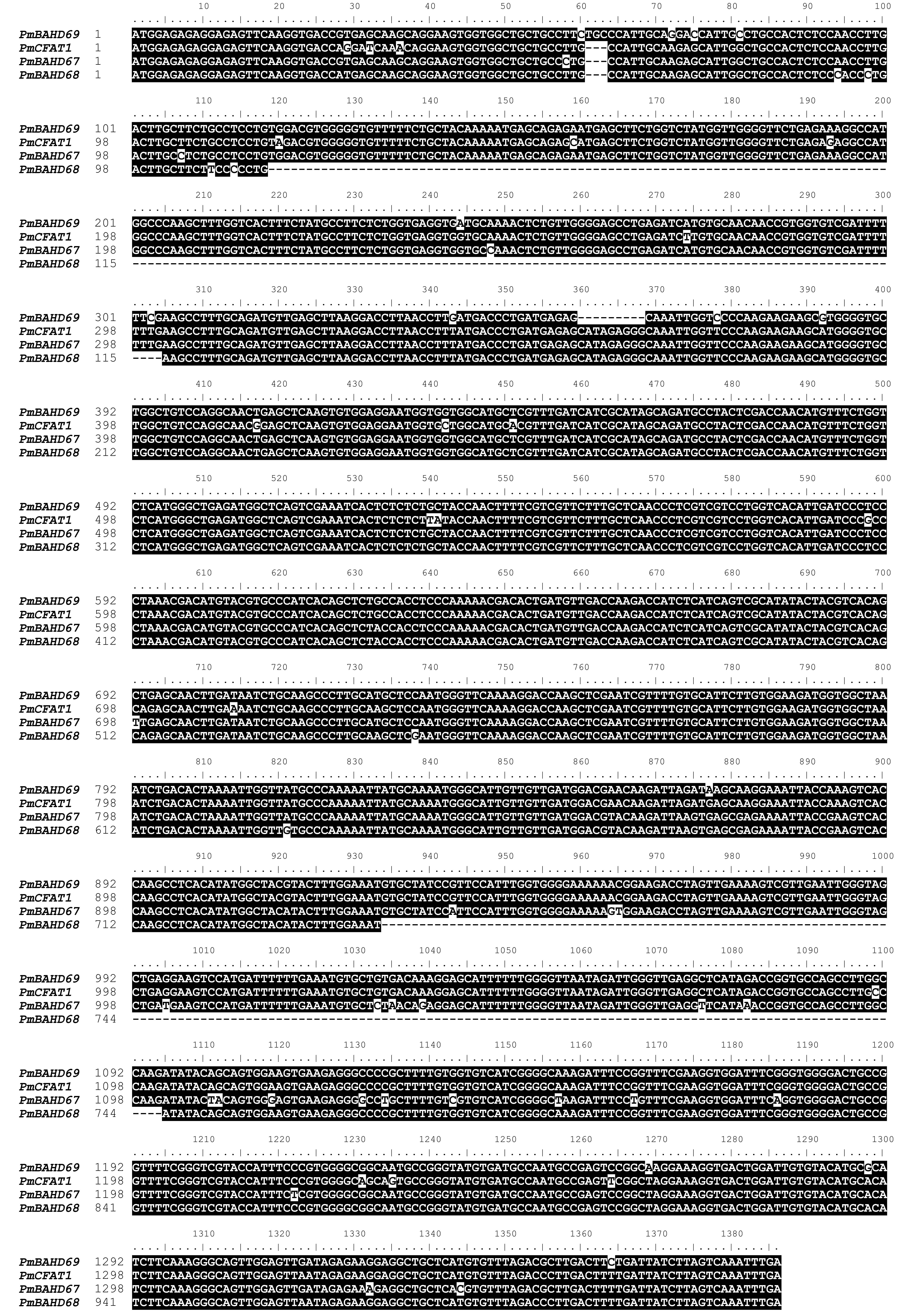

Supplement: S3 Fig — (TIF) [file pone.0223974.s004.tif]

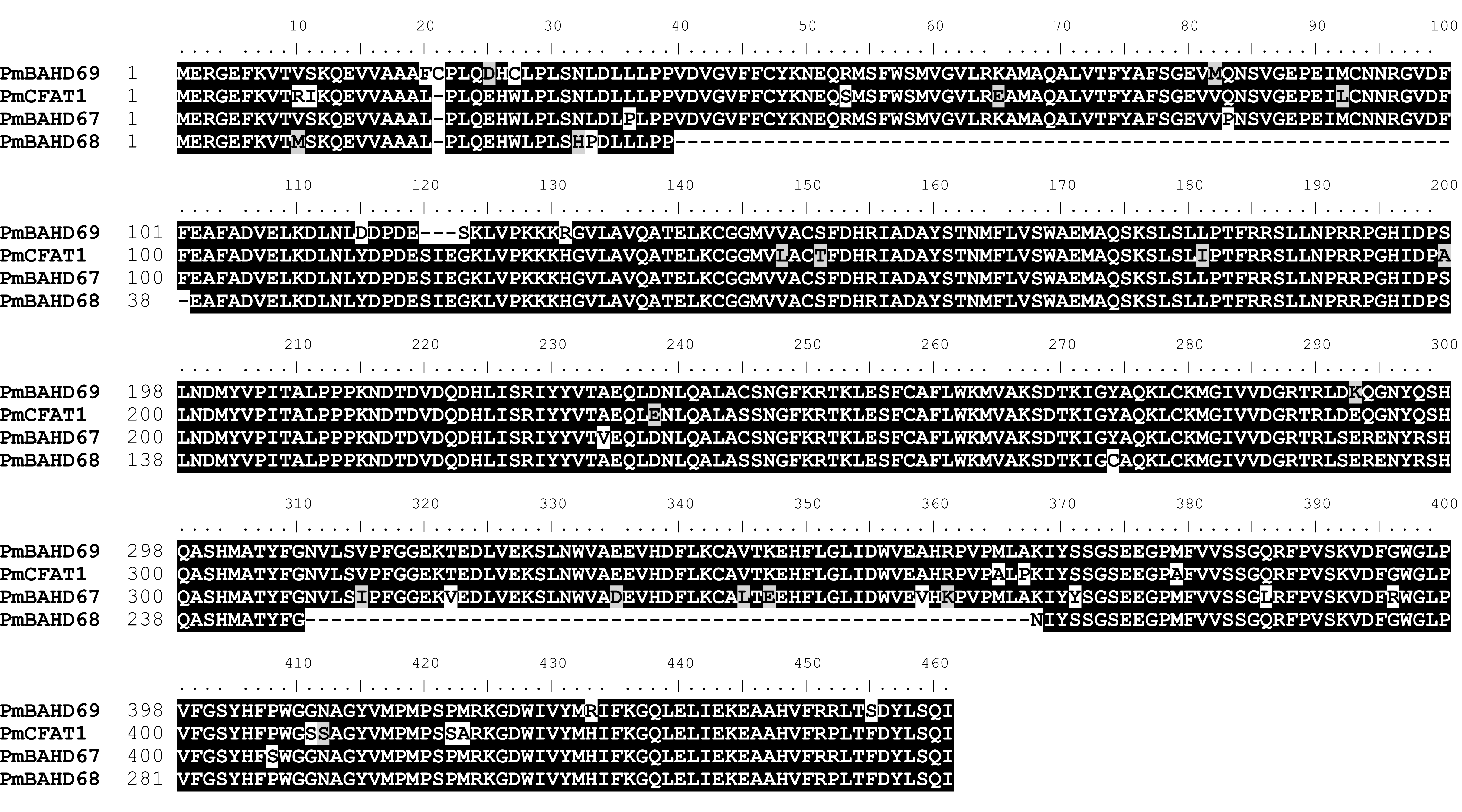

Supplement: S4 Fig — (TIF) [file pone.0223974.s005.tif]

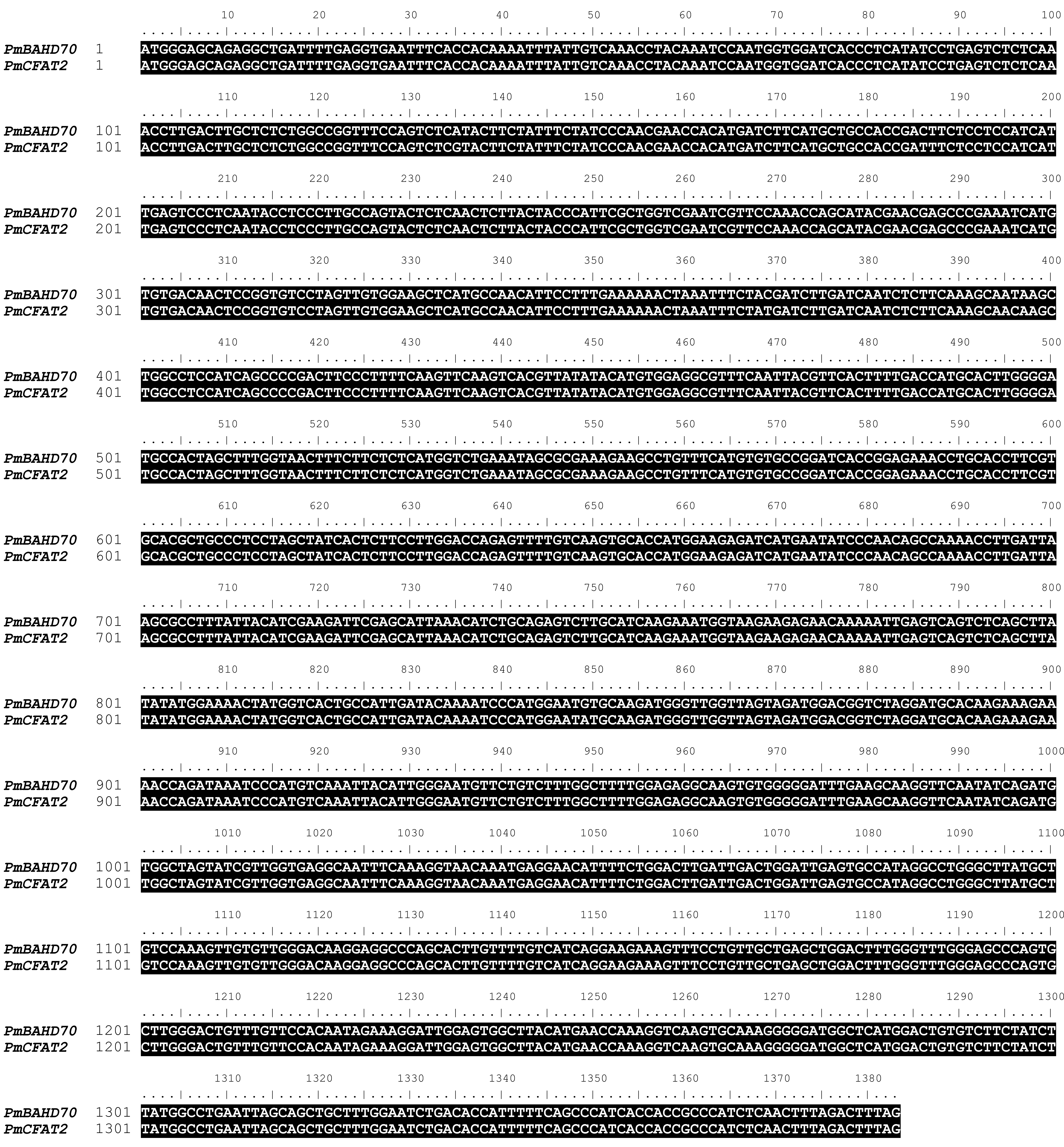

Supplement: S5 Fig — (TIF) [file pone.0223974.s006.tif]

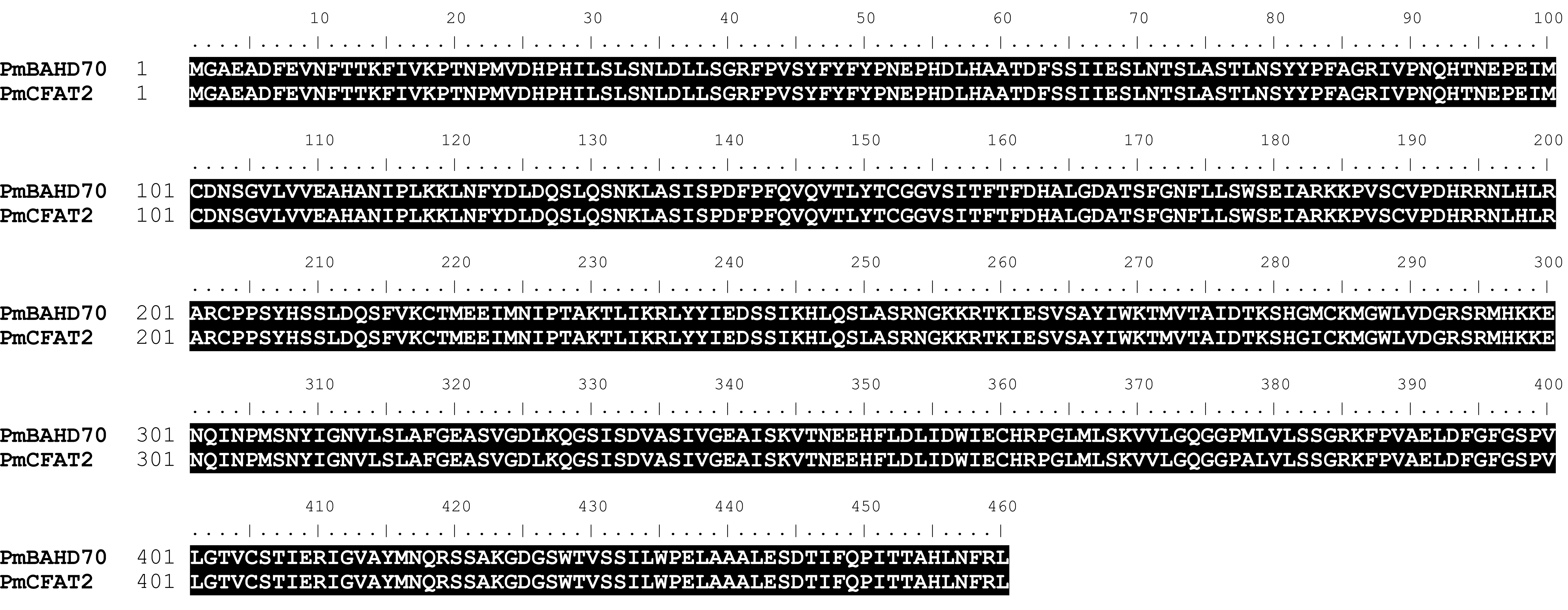

Supplement: S6 Fig — (TIF) [file pone.0223974.s007.tif]

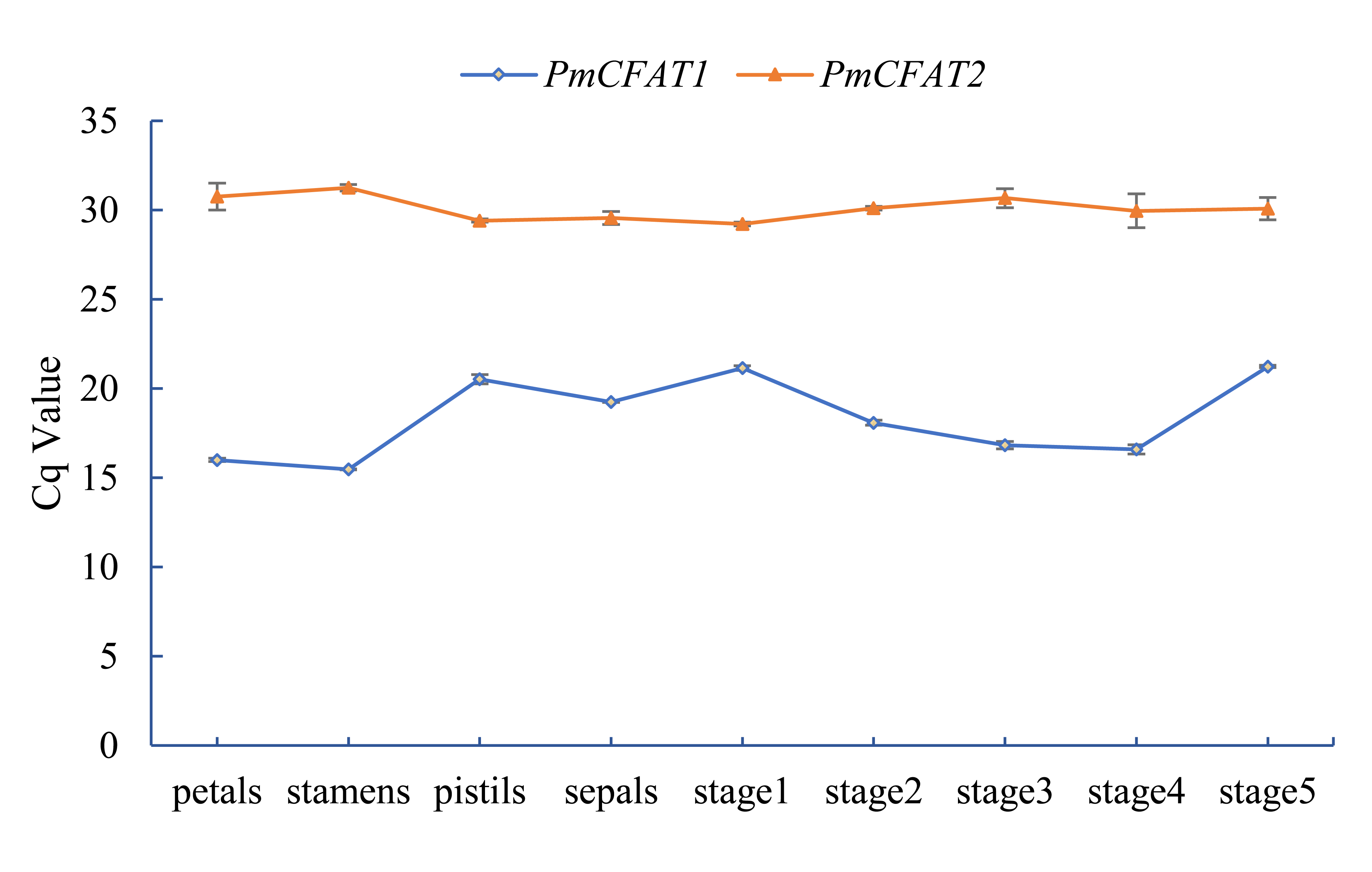

Supplement: S7 Fig — (TIF) [file pone.0223974.s008.tif]
